# Vocabulary: Common or Basic?

**DOI:** 10.3389/fpsyg.2021.730112

**Published:** 2021-11-15

**Authors:** András Kornai

**Affiliations:** SZTAKI Institute for Computer Science, Budapest, Hungary

**Keywords:** core vocabulary, basic vocabulary, word meaning, definition, word frequency, computational lexicography

## Abstract

Neither linguistics nor psychology offers a single, unified notion of simplicity, and therefore the simplest “core” layer of vocabulary is hard to define in theory and hard to pinpoint in practice. In section 1 we briefly survey the main approaches, and distinguish two that are highly relevant to lexicography: we will call these common and basic. In sections 2 and 3 we compare these approaches, and in section 4 we point the reader to Kolmogorov complexity, unfamiliar as it may be to most working psychologists, lexicographers, and educators, as the best formal means to deal with core vocabulary.

## 1. Background

Researchers and educators have a clear intuitive sense of text simplicity, and there appears to be complete agreement that simplicity is a strong contributing factor in mastering the reading task for low literacy readers, both non-native speakers and normal language learners (Watanabe et al., 2009; Paetzold, 2016); and for people suffering from language disorders such as autism, aphasia, or dyslexia (Parr, 1993; Evans et al., 2014). Unfortunately, neither linguistics nor psychology offers a single, unified notion of simplicity, and therefore the simplest “core” layer of vocabulary is hard to define in theory and hard to pinpoint in practice (Borin, 2012). Standard measures of simplicity, also known as “readability formulas” such as SMOG, F-K, Dale-Chall, etc. (see Zamanian and Heydari, 2012 for a recent survey) tend to concentrate on easily, automatically measurable factors such as the length of words and sentences.

Beyond sentence length, greater emphasis on syntactic complexity caused by the use of coordination, subordination, pronominalization, passive voice, and relative clauses, is a relatively recent area of research (Alva-Manchego et al., 2020). In this paper we will concentrate on the contribution of the vocabulary, taken to include morphological complexity as well, at the expense of syntactic measures. This is justified both by pure information-theoretic considerations (Kornai, 2019) and by functional Magnetic Resonance Imaging (fMRI) studies (Fedorenko et al., 2020).

We begin with a general survey of approaches to simplicity in the physical, biological, computational, psychological, and cognitive sciences, especially as language can be investigated from all these viewpoints. The most general approach to simplicity is to set up a scale with polar opposites ‘simple’ and ‘complex’, and some measure of where a particular entity falls on that scale. Since the basic scheme of scalar comparison is common to all these approaches, the difference must be lodged in the measure itself, and our list concentrates on these. The main variants are as follows.

**1. Ordinal measures:** Perhaps the single most popular measure in psychology and survey research is the Likert scale, typically 5 points which in our case would be “very simple, simple, neither particularly simple not particularly complex, complex, very complex,” but more detailed (7, 9, or 11 point) scales are used quite often, and Pearse, 2011 concluded that even 21 points could be helpful to the researcher. All speakers of English know that *chew* is simpler than *masticate*, and it is this knowledge that a Likert-style survey brings to light whatever granularity we impose. The subjective ‘intuitive’ sense that we spoke of above is real, and this is the method to quantify it. We may try to model this knowledge in terms of other factors (e.g., Anglo-Saxon or Latinate origin of words), but surveys are a fundamental data source, akin to grammaticality judgments, in that they constitute the explicanda for an entire field.

**2. Counting:** The assumption that entities with fewer parts are simpler than those with more parts can be traced back to the very beginnings of philosophy, the pre-Socratics. Democritus is very clear that atoms are the simplest possible things, and Plato's *Theaetetus* where knowing the wagon is equated to knowing its parts implies an epistemic commitment to a counting approach. Counting, length in particular, plays a huge role in readability formulas.

**3. Developmental measures:** Entities that appear earlier in the course of development, be it ontogenic or phylogenic, are considered simpler than those that appear later. In language development, this idea has to be taken with a grain of salt, as there are highly complex entities that appear very early on just because they are very frequent/salient. Clearly, the form *mama* correlates with a far more complex and dynamic collection of sense data, spanning more modalities, than the form *light*, whose meaning will therefore be simpler on any account, yet no infant learns *light* before *mama*.

**4. Algorithmic complexity, description length:** When component parts are not simply listed, but come with observable token frequencies/probability weights, we treat the uniform (equiprobable) distribution as the most simple, and treat additional parameters as additional complexity. This naturally leads to Kolmogorov complexity (Li and Vitnyi, 1997) and the strongly related Minimum Description Length (MDL) paradigm (Rissanen, 1978; Vitanyi and Li, 2000), which favors the shortest model over equally descriptive longer ones. Anticipating our conclusions somewhat, the analysis provided in sections 2 and 3 will furnish the empirical basis for our overall conclusion presented in section 4, that it is only this theory that can provide the right conceptual underpinnings for dealing with core vocabulary.

Historically, work on readability is closely tied to (3), and started with finding the most frequent words (Thorndike, 1921, 1931; Thorndike and Lorge, 1944) with an express pedagogical purpose, both for L1 and L2 learning. While the key assumption behind this work, that learning one word is about as hard as learning another, has stood the test of time, learnability has mushroomed into a large field of research, and even a brief overview is beyond the scope of this paper—see Klare (1974) and Paasche-Orlow et al. (2003) for informed but somewhat dated summaries, and for the more contemporary approach of bringing machine learning techniques to the task, see e.g., Pilán et al., 2014; Morato et al., 2021.

Here we take the central idea to mean simply that effort is best spent on the words that will cover the overall distribution best, i.e., on the most common ones. Remarkably, this means that serious effort needs to be spent on *function words*, because these are disproportionately present in the high frequency range. For example, in the speech portion of the British National Corpus (BNC, V2.0) that we will use in section 2 there are 9.6 m tokens for 61 k word types, and among the top 100, which jointly account for 5.6 m (58.6%) of the tokens, we find only 22 content words, half of which are prepositions. In many other languages, the same effort that in English we dedicate to function words would have to be spent on case endings and other morphological markers.

In more analytic languages like English the task of learning the grammar is intimately bound up with the task of learning the vocabulary, while in more synthetic languages like Latin the two tasks are somewhat easier to separate. Since about 85% of the information content is carried by the words (see Kornai, 2019 Ch.1.3 for discussion), comprehending them will be central to learning any language irrespective of typological differences, a fact already established in the classic (Thorndike, 1917). Importantly, recent fMRI work has established that the world's languages are functionally localized to the same brain network (Ayyash et al., 2021) so restricting this study to English does not significantly diminish the generality of our conclusions.

To fix terminology, we will refer to the frequency-based approach as aiming at *common* vocabulary, and the comprehension-based approach as aiming at *basic* vocabulary, without pre-judging the issue of how this relates to (1–4) above. At first blush, the goal of pocket dictionaries (typically 500–5,000 words) and collegiate dictionaries (typically 20–50 k) is very similar: they select a subset of the vocabulary that will provide maximum coverage in the statistical sense. This is a modern-day version of (2): we keep the word count fixed, and maximize the probability mass that can be covered by so many words^1^. It is only at the unabridged sizes that another goal, explaining what a word means, becomes evident: we look up *anaphylactic* in the dictionary precisely because it is so rare that we haven't seen it before but want to know what it means. For such an explanation to work, it is necessary to use words on the right hand side (rhs) of the definition that are, in sense (1) at least, simpler than the definiendum. We may define *masticate* via *chew*, but not the other way round, even though the two words are synonymous.

The largest contemporary effort focusing on explaining everything in simple terms is the Simple English Wikipedia https://simple.wikipedia.org, based on the principles of Ogden (1930), though not entirely faithfully (Yasseri et al., 2012). Ogden selected 850 basic words: 600 nouns, 150 adjectives, and a 100 verbs “which put the others into operation and make them do their work in statements.” His method of selection was reductive, eliminating words such as *puppy* as long as *young* and *dog* were available. In this example, both words on the rhs are simpler (1). This, as we shall see here, is not fully sufficient: we also need to guarantee that the method of combining the elements that appear on the rhs is also simple.

In this particular case the method of combining *young* with *dog* is conjunction, obviously an elementary step, but let us inspect how Webster's 3rd (Gove, 1961) deals with *anaphylactic*: ‘of, related to, affected by, or accompanying anaphylaxis’. Certainly at this stage the reader has gained very little comprehension. The true import of this definition, that *anaphylactic* is the adjectival form of *anaphylaxis*, is accessible only to the linguistically sophisticated reader—all that ordinary users see is that they must look up this other word. When they do, they find

hypersensitivity (as to foreign proteins or drugs) that is marked by a tendency to intense systemic reaction and that results from specific sensitization following one or more usu. parenteral contacts with sensitizing agent and seen chiefly in experimental animals but manifested in man in acute serum sickness and in severe or fatal reactions to later administrations of certain drugs (as penicillin).

This is hardly reassuring. Even if we ignore the difficult words and phrases (senzitization, parenteral contact, sensitizing agent, serum sickness, …), for which the reader will have to consult the dictionary again and again, substituting this rhs is the earlier definition to obtain ‘of, related to, affected by, or accompanying hypersensitivity (as to foreign proteins …’ is nontrivial. Is it *of* ? Is it *related to*? Is it *accompanying*? Is it *affected by*? All the above?

To genuinely aid comprehension, the dictionary must make the rhs simpler (1) than the definiendum, and must also guarantee that by iterated lookup this property is preserved. A major step in this direction is to restrict the rhs to a basic stratum, and to avoid the need for iterated lookup by strictly enforcing this restriction, as was done in the Longman Dictionary of Contemporary English (LDOCE, Procter, 1978). But even LDOCE permits a single indirection step (e.g., *deprecatory* is defined with the aid of *apologetic*) and gives no guidelines as to the necessary syntactic changes that must accompany such a substitution. For example, *Saturn* is defined as “the PLANET which is 6th in order from the sun and is surrounded by large rings” and at *planet* we find “a large body in space that moves round a star, esp. round the sun.” But if we mechanically substitute this rhs back in the definition of Saturn, we obtain “the a large body…” rather than “the large body….” Humans of course eliminate one of the colliding articles “the a” as a matter of course, but for a computational system the changes such substitutions trigger are not at all trivial.

Besides the Ogden list, and many other concept lists that share the explanatory goal of basic vocabularies (see List et al., 2016 for a modern system that unifies many), there is another important source for the basic approach, sometimes with overt claims for simplicity (3), but more often with the goal of uncovering genetic and areal relationships among languages. Perhaps the best known are the Swadesh (1950) and Swadesh (1955) lists, widely used in glottochronological studies to this day. Instead of “foundationality” in the sense that in principle every other word should be explained based on them, the Swadesh lists aim at “accessibility” in the sense that words corresponding to the concepts in question should not be too hard to identify in any language. There is no life without water, so words for *river, lake*, or *swim* are likely present. A quarter of the Swadesh list is devoted to natural objects, natural phenomena, and body parts, but if our goal is to define other words it is entirely inadequate. Consider the word *random*. Using the Longman defining vocabulary, we have “happening or chosen without any definite plan, aim, or pattern.” None of the rhs words appear on the Swadesh list, and it is not even clear how we could build definitions of them.

## 2. How Common Is Basic?

Here we compare vocabulary lists based on these two approaches both to see what they have in common and to uncover the salient differences. Since spoken language precedes written both ontogenically and phylogenically, we will use only the spoken segment of the BNC. While contemporary English discourse often revolves around culture-specific issues that have no direct counterpart in other languages and cultures, this is still a better proxy for approximating less resourced languages and pre-literate usage than other major corpora based on written materials.

To avoid the issue of function words, we remove the most frequent 100 of these. In speech, this list includes 17 terms that are either filled pauses *mhm erm mm ah Er Mm er*; clearly phatic *actually alright bloody look okay quite really yeah*; or both *oh ooh*. The possessive suffix '*s* is tokenized separately by the Stanza NLP package^2^ we used in the analysis, leaving us with 82 ordinary function words (see Appendix A). We also remove from the frequency count the six most common punctuation marks.,?!-; because these, in keeping with the convention that is standard in computational linguistics, are treated by Stanza as separate tokens. In total, frequent function words and punctuation are responsible for 60.6% of the tokens, with filler and phatic elements constituting 3.6%, and punctuation 11.9%. In what follows, all percentages refer to the remaining 39.6% (4.5 m tokens) of content words as 100%, though more rare function, phatic, and punctuation tokens are still present in small numbers.

We will consider five basic lists. Of these, the most ambitious is the natural semantic metalanguage (NSM) list (Goddard and Wierzbicka, 2014), in that it contains very few words, yet aims at being fully foundational, in principle offering a basis for defining every word sense in every language by combinations of a few dozen semantic primitives. In section 4 we will look more closely at the definition of *soul* offered in a cross-cultural case study (Wierzbicka, 1989). The Swadesh list already uses word combinations to distinguish word senses, e.g., *right* ‘correct’ versus *right* ‘side’, but what is a rather arbitrary disambiguation device for Swadesh, becomes a central organizing principle of NSM, which employs a variety of sophisticated syntactic constructions to define new phrases using the word list.

Next comes the (Swadesh, 1955) list, which would have good resources for function words: 22 of out of our 100 function words are listed by Swadesh, comprising 10.6% of his list. To obtain comparable numbers across basic vocabularies, we remove these here, even those two, *right* and *say*, which were clearly intended by Swadesh in the contentful, rather than the phatic sense. This is not to say that phatic skills are irrelevant for (early) language development, but corpus linguistic resources to study the issue are sadly lacking, especially as transcriptors have a strong tendency to normalize much of this out of the written corpora—studies such as (Bazzanella, 1990) are few and far between.

The 4lang defining vocabulary (Kornai, 2022) is a medium-size vocabulary (732 words, see Appendix B) aiming both at foundationality and at controlled syntax: definitions are written in a language that has its own formal grammar (and yacc parser) that regulates the manner in which elements can combine. It was obtained from the earlier (Kornai, 2019) 4lang list by systematic removal of word senses definable in terms of the remaining elements (Ács et al., 2019).

Another medium-size list is Basic English. After removal of stopwords, there remain 799 elements. Ogden (1944) was very cognizant of the differences between ordinary language use and the use of specialist vocabulary: by design, Basic English requires an additional 100 words of General Science, and 50 from each discipline he considered (physics/chemistry, geology, mathematics/mechanics, biology, business, economics). Limitations of the basic vocabulary in expressing the meaning of specialist words will be discussed in section 3.

Our last example of a basic system of words is the Longman Defining Vocabulary (LDV), 2,112 items once the function words are removed. This is the only list that is actually proven to have the power to act as foundation: LDOCE defines over 82k word senses, and there is little doubt that in a larger dictionary the authors could go further. Actually, the core LDV also contains a fair amount of (not always productive) English morphology: the prefixes *counter- dis- en- fore- im- in- ir- mid- mis- non- re- self- un- vice- well-*; and the suffixes *-able -al -an -ance -ar -ate -ation -dom -ed -ee -en -ence -er -ery -ess -est -ful -hood -ible -ic -ical -ing -ion -ish -ist -ity -ive -ization -ize -less -like -ly -ment -ness -or -ous -ry -ship -th -ure -ward -wards -work -y*. Stanza detects inflection (-s, -ind, -ed, -en) even in irregular cases like *go/went*, which makes the coverage statistics presented in Table 1 more realistic.

**Table 1:** Coverage of basic vocabularies.

**List**	**Size**	**W/o fw**	**Weight (%)**	**Avg wt (%)**	**Density (%)**
NSM	78	53	13.3	0.251	41.0
Swadesh	207	185	15.7	0.085	30.9
4lang	732	714	31.2	0.044	45.9
Ogden	850	799	33.4	0.042	48.1
LDV	2,190	2,112	64.4	0.030	78.7
∪	2,390	2310	68.5	0.030	82.7
∩_3_	464	428	30.4	0.071	50.0
UG5	1,000	913	61.7	0.068	86.5

*The first column is the original size, the second gives the size after removal of function words. Weight is the probability mass of content tokens in the BNC spoken section. See text for the last two columns*.

In addition to the five original lists, we considered their union (∪), and those that appeared in at least 3 of the 5 (∩_3_). These are not intended as a lexicographic proposal to somehow synthesize a better list: obviously the union is redundant as a basic list, and the foundationality of the majority intersection is not guaranteed. That said, they will be useful in drawing out some conclusions. The UG5 (Up-Goer Five, an XKCD comic by Randall Munroe) list, used as basic but derived as common^3^ is deferred to section 3.

First, the larger a list the better the coverage: at 2,112 content words the LDV already takes care of about 2/3 of content tokens in the spoken part of the BNC. Since the basic lists were not designed by Thorndike's methodology, this cannot simply be attributed to ‘skimming off the top’ of the Zipf distribution, but the tendency is clear for growing lists sizes. The last column of Table 1 shows the ‘density’ of a list, which shows how much of the weight that could maximally be captured by the top *n* elements is actually captured. Compared to the coverage offered by the most frequent 53 or 185 elements, the actual NSM and Swadesh lists cover only about 30–40% of the best attainable probability mass. For the medium-size 4lang and Ogden lists, density is higher: these capture about 45-48% of what a common list of the same size would have captured. Finally, a relatively large list like the LDV or the union of the five lists is almost as good as a frequency list, capturing 79% of the theoretical maximum. This number is all the more remarkable given that the UG5 list, which was obtained on a different corpus of English by simply taking the top 1,000 (of which we ignore the function and phatic elements) gets only 86.5% on the BNC spoken materials.

Second, the smaller the list the more general the terms. Even the rarest terms in NSM, *below* and *above*, occur several hundred times each. In contrast, LDV contains 1,146 terms that occur less often than any of the NSM terms, including several like *admittance, adverb, gasoline* that occur only once in the spoken BNC, and some like *cowardly* or *nobleman* which do not occur there at all. The next to last column of Table 1 shows the average contribution of a list word to the probability mass. The more basic a list, the larger this average contribution turns out to be, indicating not so much the selection of high frequency words as tighter control in terms of excluding really low-frequency ones.

## 3. How Basic Is Common?

In a broad sense, the results of section 2 vindicate both Thorndike and Ogden. Proponents of Thorndike's approach could say: just get the first 1,500 most frequent words, and you covered all the basic vocabulary, since if you covered the NSM list you are done. Proponents of Ogden's approach could say: that is really wasteful, you are using a 1,500 words to accomplish something you could get done by a few dozen.

The pedagogical concern of Ogden and Thorndike is evident, but neither of them could have anticipated how much the goalposts have moved. Today, our interest is not just with L1 and L2 learners, but also with computers: a clear goal of AI, first set by Turing (1950), is to have intelligent conversations with machines. We aim at far more than the ability to deceive a human (Shieber, 2007), the custom-designed Winograd challenge (Levesque et al., 2012) and the updated WinoGrande challenge (Sakaguchi et al., 2020) exercise many semantic facilities. For readers not familiar with this work, here is a typical paired test question:

The large ball crashed right through the table because it was made of styrofoam *What was made of styrofoam, the ball or the table?*The large ball crashed right through the table because it was made of steel. *What was made of steel, the ball or the table?*

In addition to the obvious grammatical prerequisites, the task exercises not just encyclopedic knowledge (steel is hard, styrofoam is fragile), but also a generic conceptual scheme, that normally it is hard things that crush through fragile ones and not the other way round.

To see how well common vocabulary can be used to define specialist words, we will briefly survey the 300 entries offered in the spirit of Randall Munroe's *Up Goer Five*^4^ explaining terms like *syntax* using “only the 1,000 words people use the most often.” The thousand most frequent words were derived from written sources, the Wiktionary contemporary fiction frequency list,^5^ and as such, it is well resourced in function words (covers 82 of our 100), but far from ideal for content words (86.5% density, see the last line of Table 1). Since morphology is largely taken care of by the Automatically Generated Inflection Database,^6^ in principle the UG5 vocabulary could work well for explaining technical work such as summarizing PhD theses and for defining specialist words. But there are several recurring problems.

First, the use of *idiomatic English*. Consider “…interesting because that gives us a real leg up in finding out how the mind works”—readers unfamiliar with the English idiom *to give a leg up* will not be able to figure out what is being said here.

Second, *using multiple senses*. For example, the original XKCD cartoon uses *space* both in the sense ‘the area beyond the Earth where the stars and planets are’ and, for a helium pressurization tank described as “more funny voice air (for filling up space)” in the sense ‘the amount of an area, room, container etc that is empty or available to be used’.

Third, *associative descriptions*. “funny voice air” works well as an associative hint for helium, at least for those familiar with helium speech. “the kind of air that once burned a big sky bag” also works well for hydrogen, but only for those aware of the Hindenburg disaster.

Fourth, *nonce compounding*. With a bit of luck, everybody can figure out that “train-food” means fuel. But what are “idea-paper, air-light, pretend-box” or “fire rock”?

Fifth, *circumlocution*. We may be able to figure out that “a jumping animal that lives in the water and makes noise” is a frog (even though frogs don't live in water), but what is “the stuff that comes out of the animal with white and black spots”?

Sixth, *lack of naming*. A very large proportion of the specialist vocabulary refers to technical concepts that have a reserved meaning or directly reserve (create) a new meaning for a non-technical term. To learn about liquid oxygen “cold air for burning” we first need to learn about liquefying and fractioning gases: “wet and very cold” air would mean something entirely different in everyday language.

The first two problems are easily remedied by a system that does more than mechanically check the description against a word list. The third one actually leverages the preexistence of the kind of world knowledge that it aims at creating. Actually, nonce compounds and circumlocutions have the same mechanism, when they work, and they fail precisely when the outside knowledge is for some reason hard to access.

Ogden's approach was to leave room for 50 specialist words in each field of science he considered. Unfortunately linguistics, psychology, or cognitive science was not one of them, and for this reason we also omitted the specialist vocabulary of 4lang, which includes grammatical terms like *agent, patient, instrument, …* and logical terms such as *cause, part-of, …* since these are never used in the BNC in the technical sense.

## 4. Conclusions, Further Work

In the final analysis, we see lack of naming not as a problem but as a solution. For an example from the same corpus^7^, consider the following: “Everyone knows how to add numbers together. Right? But sometimes we want to use things that are not numbers and that is hard. We wish we were adding numbers instead. So we came up with a thing called a “group”. We wrote down all the things that numbers do when you add them. And we said: if something does all the things that numbers do when you add them, then that thing is a “group” …”

Once we permit definitions, we may really begin to explain things. Everyone knows how to add numbers together. Right? This is called *addition*. But sometimes we want to use things that are not numbers and that is hard. This is called *symbolic computation*. So we came up with a thing called a “group”. We wrote down all the things that numbers do when you add them. These are called *group axioms*. And we said: if something does all the things that numbers do when you add them (this is called *satisfying the group axioms*), then that thing is a “group”!

If things can be named, we are able to do away with the puzzle-solving aspect entirely, except for natural kinds (Quine, 1969). The fact remains that one either knows that the “animal of central Asia that looks like a cow with long hair” (LDOCE) is a *yak* or one can accept this as the definition of ‘yak’, there being no competing central Asian animal that would fit the rest of the definition. Once you have *milk* defined as “a white liquid produced by cows or goats that is drunk by people” (LDOCE), you no longer need to play clever games about the animal with white and black spots. The humorous effect of the original Up Goer Five comic and the subsequent *1,000 words of science* entries lies in great part in the puzzle-solving, but if our goal is actually to convey information, especially to those who don't already have it, *adding recursive definition of new words and phrases is a must*.

On the whole, when we speak of simple language, we generally mean both simple vocabulary and simple grammar. Here we concentrated on vocabulary, offering only a few tentative remarks in regards to grammar. Yet it is clear that to a certain extent these two are fungible: we can tighten the vocabulary at the expense of longer definitions. As an example, let us consider the NSM definition of a cross-culturally salient, albeit non-scientific concept, *soul*. (Line numbering added to the original definition in Wierzbicka, 1989, p 43.)


1. one of the two parts of a person
2. one cannot see it
3. it is part of another world
4. good beings are part of that world
5. things are not part of that world
6. because of this part, a person can be
a good person


Notice the syntactic complexities in this definition. By using *the*, (1) already presupposes a theory: a person has two parts. The soul is one of these two. To simplify matters, we will call the other, which remains unnamed throughout the definition, the body, especially as this word is already part of the very limited NSM vocabulary. (2) is a simply conjoined statement that the soul is invisible, not any different from any ordinary definitional clause, e.g., that glass is transparent, or elephants are large. (3), however, introduces a new entity, another world, which again comes with an existential presupposition of there being one (ordinary) world relative to which this world counts as “another.” (4) and (5) serve to define the other world, and we note that it takes a great deal of syntactic sophistication to recover the that world of these clauses as the *other world* of clause (3), while this part in clause (6) is resolved as the definiendum *soul*.

Also implicit in the definition is some general compilation of things, a *world*. (This is problematic only because we don't have an NSM dictionary of English.) The point to be noted is that we see the same generic conceptual scheme one–other invoked twice: once for parts of a person, and the second time for worlds. We have argued elsewhere that this conceptual scheme is tied to the meaning of *other* (see Kornai, 2022 Figure 1.3) but whatever solution one might propose, it takes significant discourse representational resources (Kamp, 1981; Heim, 1982) to keep these two instances separate.

Syntactic complexities aside, this is remarkably close to the LDOCE definition of *soul* “the part of a person that is not physical, and that contains their character, thoughts, and feelings. Many people believe that a person's soul continues to exist after they have died,” which also accounts for the doctrine, seen in many religions, of the immortality of souls (but does not make this an essential feature of the definition). The underlying theories are also similar in asserting the non-physical nature of the soul, and in positing it as the locus of goodness (character). In fact, LDOCE offers a different sense ‘the special quality or part that gives something its true character’ as in *Seafood is the soul of Provencal cousine*.

To summarize, a notion of *core* vocabulary that is useful for psychologists, linguists, and educators alike must synthesize the definitional simplicity (basic) and the high occurrence (frequent) aspects. Of the approaches we surveyed in section 1 it is only (4), Kolmogorov complexity, that is capable of doing this. To guarantee fungibility, we will say that the complexity of a defined term such as *group* will be equated to the complexity of its definition “a thing that satisfies the group axioms.” This way, introducing and using defined terms incurs no extra penalty. To make shorter definitions simpler, we use a counting measure (2) that counts all the primitives at the same unit value. We also add a coordination penalty *c* to various clauses, roughly speaking by counting the commas in the definition.

There remain several important questions for further work. Do we wish to count conceptual schemas, such that *other* presupposes *one*, or that hard things crush fragile things, as part of some lexical entries, or do we amortize these over many instances where they are used? How do we count the complexity of function words and bound morphemes, entirely ignored in this study? The answers are of necessity tied to the model of syntax and morphology chosen, and unless we make strides in universal syntax and morphology, we may have to rely on language-specific stopgap measures.

## Data Availability Statement

The raw data supporting the conclusions of this article is made available by the authors at https://kornai.com/VCB.

## Author Contributions

The author confirms being the sole contributor of this work and has approved it for publication.

## Funding

This work was supported by MILAB, the Hungarian artificial intelligence national laboratory.

## Conflict of Interest

The author declares that the research was conducted in the absence of any commercial or financial relationships that could be construed as a potential conflict of interest.

## Publisher's Note

All claims expressed in this article are solely those of the authors and do not necessarily represent those of their affiliated organizations, or those of the publisher, the editors and the reviewers. Any product that may be evaluated in this article, or claim that may be made by its manufacturer, is not guaranteed or endorsed by the publisher.

## Footnotes

^1^For historically accuracy we should mention that Thorndike's *Word Book*s actually attempted to balance considerations of text frequency (TF) and document frequency (DF), really aiming at maximum coverage over a variety of genres, anticipating (Spärck Jones, 1972) to a remarkable extent.

^2^ https://stanfordnlp.github.io/stanza

^3^ https://splasho.com/blog/2013/01/17/

a-bit-more-about-the-up-goer-five-text-editor

^4^ https://xkcd.com/1133

^5^ https://en.wiktionary.org/wiki/Wiktionary:Frequency_lists/Contemporary_fiction

^6^ http://wordlist.aspell.net

^7^ https://tenhundredwordsofscience.tumblr.com/archive
