# Supplementary material for: Vocabulary: Common or Basic?
Source: Front Psychol. 2021 Nov 15;12:730112. doi: 10.3389/fpsyg.2021.730112 (PMC8634872; doi:10.3389/fpsyg.2021.730112)
Supplement: Supplementary file 1 [file Data_Sheet_1.pdf]

Supplementary material for *Vocabulary: common or basic?*

Appendix A: BNC function words The top 100 function words in the Stanza-analyzed BNC speech corpus. Fillers and phatic elements are in *italics*.

's I *Er Mm* a about *actually ah alright* also and any anyway as at aye be because *bloody* but by can cos could do else *er erm* even for gonna gotta have he how if it just like *look* may mean *mhm* might *mm* must my no not now of *oh okay* one only *ooh* or people perhaps please *quite* rather *really* right say she should so some somebody something sorry still sure tell than that the they thing this through to too very we well what when where whether which who why will with would *yeah* yes you

Appendix B: `4lang` The `4lang` defining vocabulary. Function and phatic words are in *italics*

-able -est -ize -er -ing -ist -th Asia acid act action activity add after aggressive agriculture aim air alcohol all allow amount anger angry animal area arm around artefact ash *at* atmosphere atom attack attract authority autumn awake axis bad bake bark base beam beautiful bed bee before begin belief believe below bend best between big bird bite black blade blame blood blue body bone border bottom book bowl box brain branch breath breathe bright broadcast brush building bullet burn bus business buttocks buy buyer calculate calm camera *can* car carbon care carry cause centre cereal chair change characteristic chew chicken child chin choose circle circular clean clear close cloth cloud coal cold colour column command common communicate company compete complex conduct confident conform connect connected conscious constant contact contain container control cook cool cord corner correct cotton count country court cover cow crop cube cupboard curve cut cutlery cylinder damage danger dark day death decide decorate deep defeat defend deliberate desire detail determine device diamond different difficult dimension direction dirt disease distance disturb divide *do* dog door dot down drink drive drop drunk dry dust ear early earth easy eat edge effect effort egg electric electricity element elephant emotion empty enclose end energy engine enter entrance equipment event exact exchange exist expect experience express extreme eye face fact faith faithful fall family far fast fat fear feather feel feeling female fibre fight fine finger fire firm first five fix flame flat flesh flexible floor flour flow flower fly follow food foot *for* force forehead fork form four frame frequent fright frighten from front fruit fur furniture further future gas gen get give go good govern government grain graphite great green grey ground group grow gun hair hand handle happen hard harm has head health healthy hear heat heavy height help high hill hold hole hollow home honey horizontal horse hot hour house human hurt husband

ice idea ill image imagination important in information injury insect institution instrument intense interrupt iron item ivory jaw join joint joy judge kind king knife know lack land large law lead leaf leg legal lens letter level lid life light *like* limb limit line liquid list little live long lose loud love low lung machine main make male mammal man many mark marriage mass material *mean* meaning measure meat member memory mental message metal middle milk mind mineral minute monarch money month more most motion mountain mouth move much mud muscle *must* natural near neck need needle nervous nice night noise norm normal nose notice *now* number object ocean offend official often on *one* open opinion opponent oppose *or* order organ organization organize other out outdoor outer owner page pain paint palm paper parallel parent part particle passenger past pause pay *people* perceive period person photograph phrase physical picture piece pierce pipe place plan plane planet plant pleasant point polish political politics position possibility possible powder power practice pressure price problem product programme proof protect prove public pull purpose put quality quantity queen quick quiet radio rain raise range rank read real realize reason receive recent rectangular red regular relation religion religious remember report represent reproduce resemble responsible rest rice ride rigid road roof room root rope round royal rule run sad safe salt same *say* scale screen season seat second see seed self sell seller sensation sense sentence separate sequence series set seven several sew sex shape sharp sheep sheet shelf shell shine shoot shop short shot shoulder show shut sick side sign signal similar sit situation six size skin sky slide slope small smell smooth snow society soft soil solid solve sorrow sound soup sour space speak speech split spoon spring stable stand star start state steel stem stick sticky *still* sting stomach stop store straight strong structure student study substance succeed success sudden sugar summer sun sunrise sunset support surface swallow sweet symbol system table talk tall taste television temperature temple text thick thin *thing* think *this* thought thread threaten three throat *through* thumb time tired *to* together tongue tool tooth top touch transport travel tree truck true trunk try tube turn tusk two under understand union unit upset use useful value vehicle vertical violent visible wall want warm warn water wave way weapon weather week weight wet wh wheat wheel white whole wide wife *will* win wind wing winter woman wood wool word work wrist write written year yellow young

(For downloadable files see https://kornai.com/VCB)
